# Study on Multi-Pass Steel Ball Push Bending Process of 5A02 Aluminum Alloy Thin-Walled Tube with 0.8 D Bending Radius

**DOI:** 10.3390/ma19214604

**Published:** 2026-10-30

**Authors:** Yougen Dong, Xuefeng Xu, Haoxing Xu, Yanfang Li, Yubin Fan, Jun Xie

**Affiliations:** 1 School of Aviation Manufacturing and Mechanical Engineering, Nanchang Hangkong University, Nanchang 330063, China; yougenfz@163.com (Y.D.);; 2 Demonstration Center for Engineering Training Education, Nanchang Hangkong University, Nanchang 330063, China

**Keywords:** 5A02 aluminum-alloy thin-walled tubes, diameter of the steel balls, the length of the tube blank, lubrication conditions

## Abstract

During multi-pass push-bending, the supporting behavior at process interfaces may induce microscopic surface damage and thereby adversely affect the bonding performance of subsequent surface treatments. To address the excessive cross-sectional ovality in conventional tube push-bending and the difficulty of recovering liquid filling media, this work proposes a novel multi-pass push-bending process using discrete steel balls as filling and supporting media, with 5A02 aluminum alloy tubes of Φ32 × 1 mm selected as the research object. A corresponding finite element model is established, and orthogonal simulations are performed to optimize the parameters, including steel ball diameter, tube blank length, and tube–die friction condition. The results show that a steel ball diameter close to the inner diameter of the tube blank provides stronger internal support and remarkably restrains cross-sectional distortion. A shorter tube blank facilitates material flow in the bending zone and alleviates excessive inner-wall thickening. Reducing the tube blank–die friction effectively suppresses excessive outer-wall thinning. While achieving favorable macroscopic forming quality, the proposed process also exhibits promising potential for reducing surface scratches and improving surface integrity.

## 1. Introduction

In recent years, with the growing application of novel lightweight and high-performance bent tube components, more stringent requirements have been imposed on the forming indicators of bent tubes in aerospace and automotive industries [1]. A variety of tube push-bending processes have been developed so far. Nevertheless, defects such as uneven wall thickness distribution, cross-sectional distortion, and end cracking readily occur during forming under the coupled effects of multiple factors [2,3].

Xu et al. [4] investigated the effect of polyurethane inserts on cross-sectional distortion in cold push-bending and concluded that increasing internal pressure can reduce distortion. Lin et al. [5] adopted hydroforming bending technology and verified that the variation of internal pressure can prevent cross-sectional deformation. Zhang et al. [6] applied different pressures during preforming and feeding stages to successfully control the cross-sectional profile and avoid wrinkling and fracture. Zhang et al. [7] proposed a novel hydraulic push-pull bending technique, where internal pressure is used to suppress wrinkling and cross-sectional deformation. These studies demonstrate that proper regulation of internal pressure can mitigate cross-sectional deformation of bent tubes.

To address the uneven wall thickness distribution of tube blanks during forming, Yang et al. [8] adopted an end compensation structure to mitigate material thickening on the inner bending side, together with zoned lubrication to reduce the risk of wrinkling. Xu et al. [9] studied the effect of zoned lubrication on wall thickness, and the results show that this method can effectively prevent wrinkling. Chen et al. [10] established a finite element model for stainless steel tube push-bending and carried out corresponding experiments. Their results indicate that a higher friction coefficient can reduce outer-wall thinning while aggravating inner-wall thickening. Cheng et al. [11] explored the influence of tube–die friction on bend formability via free-bending experiments and finite element simulations, proving that larger friction coefficients lead to more severe wall thickening and cross-sectional deformation. The above findings reveal that tuning the friction between tube blank and die can improve the uniformity of wall thickness distribution on both inner and outer sides of bent tubes.

Xie et al. [12] adopted a cold push-bending process with polyurethane internal expansion to solve the cross-sectional distortion of bent tubes. They found that excessive back thrust leads to insufficient forming of the tube, while insufficient back thrust causes tube instability and end distortion. Kami et al. [13] used an artificial neural network to predict wrinkling during tube bending and demonstrated that the hybrid method combining ANN and finite element simulation can accurately predict wrinkling in push-bending. Pan et al. [14] proposed a novel theoretical and numerical framework for wrinkling prediction of tubes fabricated by free bending forming (FBF). The results show that an increase in axial force weakens the anti-wrinkling capacity and raises the wrinkling tendency, and the established numerical model can effectively predict wrinkling initiation. Wu et al. [15] simulated experimental conditions, extracted servo signal features, and combined them with a random forest algorithm. They verified that servo signals coupled with the random forest algorithm can indirectly and effectively evaluate tube forming quality. Hu et al. [16] investigated the effects of temperature, extrusion stress and friction coefficient on the bending quality of AZ31 magnesium alloy tubes under the combined extrusion-continuous shear bending process. Zhang et al. [17] combined simulation and experiments to study the wall thickness evolution of thin-walled tubes during bending and hydroforming, providing a theoretical basis for wall thickness uniformity optimization of lightweight materials. Song et al. [18] proposed a new particle-medium-filled assisted push-bending method. By combining the discrete element method (DEM) with experimental validation, they revealed the mechanical behavior of particle media during push-bending and proved that the internal pressure generated by filling media can effectively suppress wall wrinkling and cross-sectional ovalization. Fu et al. [19] developed a novel hot gas pressure bending (HGPB) process for ultra-thin large-size TC4 titanium alloy tubes. Internal gas pressure and thermal effects are adopted to adjust the stress state during bending, which effectively suppresses wrinkling and preserves cross-sectional integrity. Taghizadeh et al. [20] studied rotary draw bending of pure titanium tubes with steel-ball filling and resistance heating. This process relies on thermal softening together with steel-ball support to improve filling stability and suppress wrinkling and cross-sectional distortion of thin-walled tubes. Nevertheless, their work focuses on single-pass hot rotary draw bending rather than multi-pass staged cold push-bending, so it cannot be directly applied to achieve ultra-small cold bending with R/D = 0.8.

Apart from experimental and numerical simulation approaches, the cross-sectional ovalization of thin-walled tubes during bending has also attracted continuous attention from theoretical analytical investigations. Iandiorio et al. [21] adopted the tension field theory to systematically analyze the mechanical response of internally pressurized bent tubes before and after wrinkling. They revealed the evolution of cross-sectional flattening and multi-wavelength wrinkling modes of bent thin-walled tubes under the Brazier effect, establishing a theoretical framework for the coupled prediction of cross-sectional distortion and buckling under internal pressure. Pascal-Abdellaoui et al. [22] proposed a hybrid shell-beam element model that couples beam kinematics with shell-type cross-sectional displacement fields. Circumferential Fourier expansion is used to characterize the nonlinear coupling between local cross-sectional deformation and global rotation, which substantially reduces computational cost while retaining the accuracy of shell theory for cross-sectional deformation. Recently, aiming at cross-sectional deformation of thin-walled components under large displacement conditions, Pastrana et al. [23] developed a shell theory considering geometric nonlinearity. This theory can describe the evolution of cross-sectional deformation and ovalization for open and closed thin-walled tubes under large-displacement bending, offering a new theoretical framework for predicting tube cross-sectional responses under complex deformation conditions. Although the above analytical models can effectively predict the ovalization behavior of bent tubes without internal support, most existing theories are developed for single-pass pure bending cases. They struggle to characterize the interaction between tube cross-sectional deformation and material flow under the dynamic action of internal supporting media and the coupling effect of multi-pass forming. Therefore, it is of great research significance to reveal the evolution mechanism of cross-sectional ovalization for thin-walled tubes in the multi-pass push-bending process coupled with dynamic steel-ball replenishment.

The foregoing studies demonstrate that various internal support methods (granular media, polyurethane inserts, steel-ball filling, hydraulic pressure, mandrels) can suppress wrinkling and cross-sectional distortion of bent tubes from different perspectives. Nevertheless, existing processes still exhibit obvious limitations for cold push-bending with an ultra-small relative bending radius of R/D ≤ 1.0.

As shown in Table 1, comparative analysis indicates that conventional and existing processes (mandrel support, granular filling, single-pass steel-ball support, etc.) are constrained by inherent physical mechanisms, and their bending limits are mostly limited to R/D ≥ 1.0. The proposed process combining discrete steel-ball support and multi-pass staged push-bending realizes continuous replenishment of internal supporting media by refilling discrete steel balls between passes, breaking the forming-limit bottleneck of existing methods and achieving stable cold bending at R/D = 0.8.

Different from the available single-pass bending technologies with steel-ball or granular internal support, the novelties of this work lie in three aspects. First, this work presents an innovative forming strategy: a four-pass staged push-bending scheme is adopted, where one drastic large deformation is divided into multiple small incremental deformations to complete tube bending gradually, so as to reduce stress concentration and cross-sectional distortion within each pass. Second, this work presents an innovative supporting mode: discrete steel balls are replenished between adjacent forming passes to sustain rigid internal support in the bending zone, overcoming the drawback that supporting media drift away with tube material flow during single-pass forming. Third, this work presents process optimization: orthogonal tests are performed to reveal how steel-ball diameter, tube blank length and tube–die friction affect wall thinning and cross-sectional oval distortion, and a multi-objective preferred parameter combination is obtained. Relying on the idea of staged medium replenishment and pass-by-pass progressive support, the proposed multi-pass discrete steel-ball push-bending process achieves high-quality push-bending of thin-walled aluminum alloy tubes at R/D = 0.8 under the present experimental conditions. It should be noted that this result only verifies the forming capacity of the proposed process within the current parameter range and does not rule out the possibility that other internal-support processes can realize the same small-radius bending after proper adaptation and optimization.

In this study, 5A02 aluminum alloy tubes with an outer diameter of 32 mm, wall thickness of 1 mm, and relative bending radius of 0.8 D (where D denotes the tube outer diameter) are selected as the research object. A finite element model with steel balls as filling media is established, and orthogonal simulations together with push-bending experiments are carried out. The effects of steel-ball diameter, tube blank length, and tube–die friction coefficient on the forming quality of multi-pass push-bending are investigated. The evolution laws of wall thickness and cross-sectional oval distortion under multi-pass forming are revealed, and the forming advantages of the multi-pass strategy with inter-pass steel-ball replenishment for cold push-bending at 0.8 D are validated. This work provides theoretical support for the engineering application of push-bending of thin-walled tubes with small bending radii.

## 2. Analysis of Push-Bending Process

### 2.1. Forming Principle

During push-bending, the contact between steel balls and the inner wall of the tube blank is point-to-surface contact, which fails to effectively improve the stress distribution on the tube inner surface. The target component is a thin-walled aluminum alloy bent tube with a relative bending radius of 0.8 D. If single-pass direct forming is adopted, the pusher has to travel a displacement far larger than the steel-ball diameter. Nevertheless, under the axial thrust F1, the steel balls located at the 45° end of the tube blank tend to slip out of the tube blank. Consequently, the unsupported extended section of the tube wrinkles without the internal support provided by steel balls. To tackle this issue, this work proposes a novel multi-pass push-bending method for tubes with small bending radii. By adjusting the push-bending parameters and steel-ball positions, the target geometry can be gradually approached, enabling the forming of thin-walled tubular components with small bending radii.

**Figure 1:**
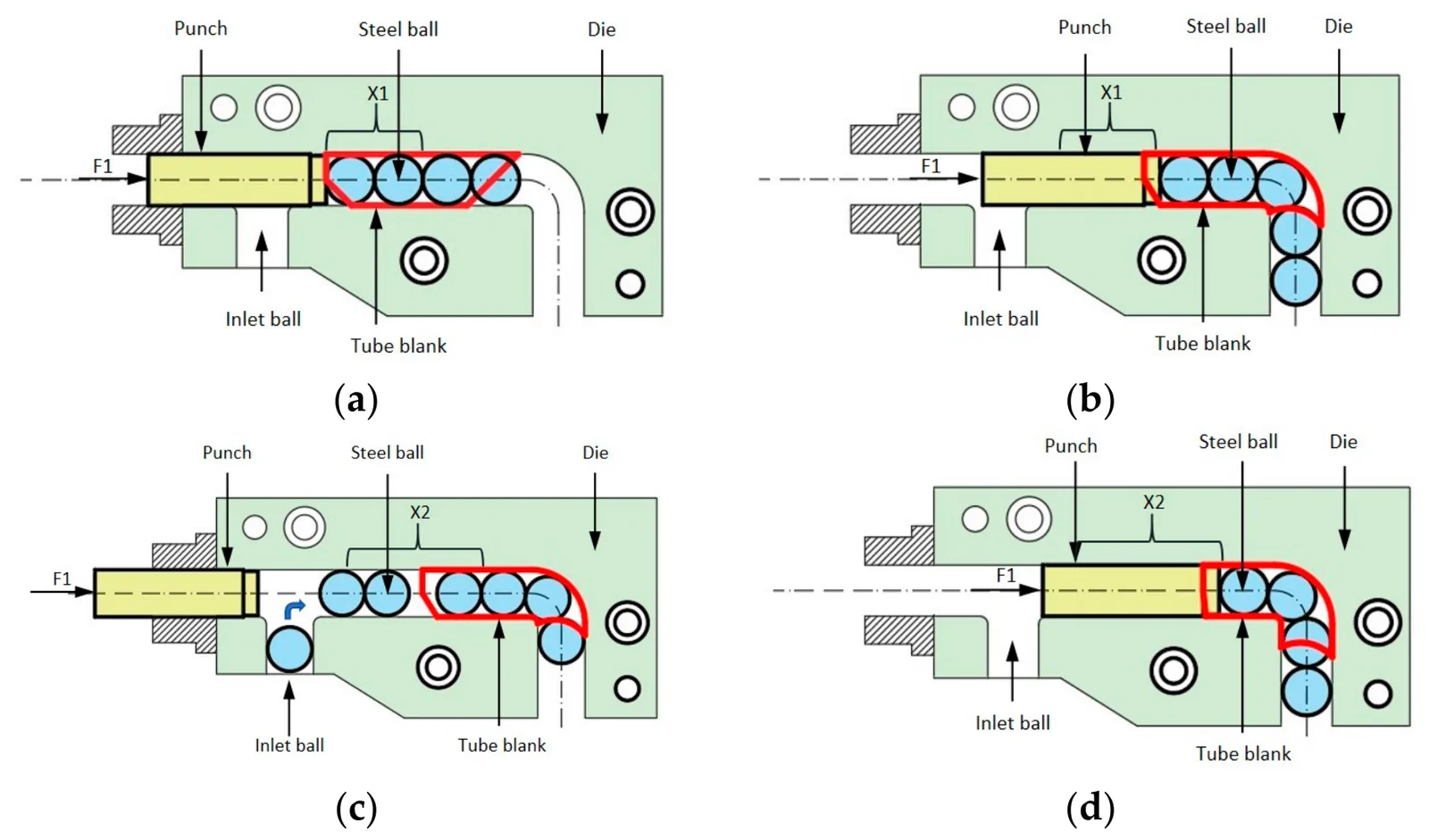
Schematic diagram of push bending forming. (**a**) Initial state, (**b**) the first pass, (**c**) insert steel balls, (**d**) the second pass.

The push-bending process is illustrated in Figure 1. The tube blank is placed inside the die and filled with steel balls, and the pusher contacts the end of the tube blank, as shown in Figure 1a. During bending, the pusher advances slowly by a displacement X1 to drive deformation of the steel balls and tube blank, as shown in Figure 1b. Afterwards, the pusher retracts to its initial position, and additional steel balls are replenished through the ball inlet, as shown in Figure 1c. In the second push-bending pass, the pusher advances slowly by a displacement X2 (X1 < X2), as shown in Figure 1d. The above push-bending cycle is repeated with tuned pusher feed displacement until the bent tube is fully formed.

Bent tubes formed by single-pass and two-pass schemes exhibit remarkable wall thickening on the inner bending side, which induces wrinkling and uneven wall thickness distribution. In addition, severe wall thinning at the bending end brings a risk of fracture. The wall thickness distribution of the bent tube formed by three passes is better than those obtained from one and two passes, yet obvious inner-side thickening and a tendency toward wrinkling can still be observed, accompanied by considerable outer-side thinning. By contrast, the four-pass bent tube achieves uniform wall thickness distribution, smooth surface and no obvious defects. It can be concluded that increasing the number of forming passes promotes a more uniform wall thickness distribution, improves forming quality and enhances the formability of bent tubes to a certain extent. Considering that the growth rate of dimensional accuracy gradually levels off as the number of forming passes rises, and more passes reduce processing efficiency, the four-pass formed part satisfies practical engineering requirements in terms of dimensional accuracy and surface quality. Therefore, no additional forming passes are adopted, and the steel-ball-assisted four-pass push-bending process is finally selected.

### 2.2. Mechanical Analysis

**Figure 2:**
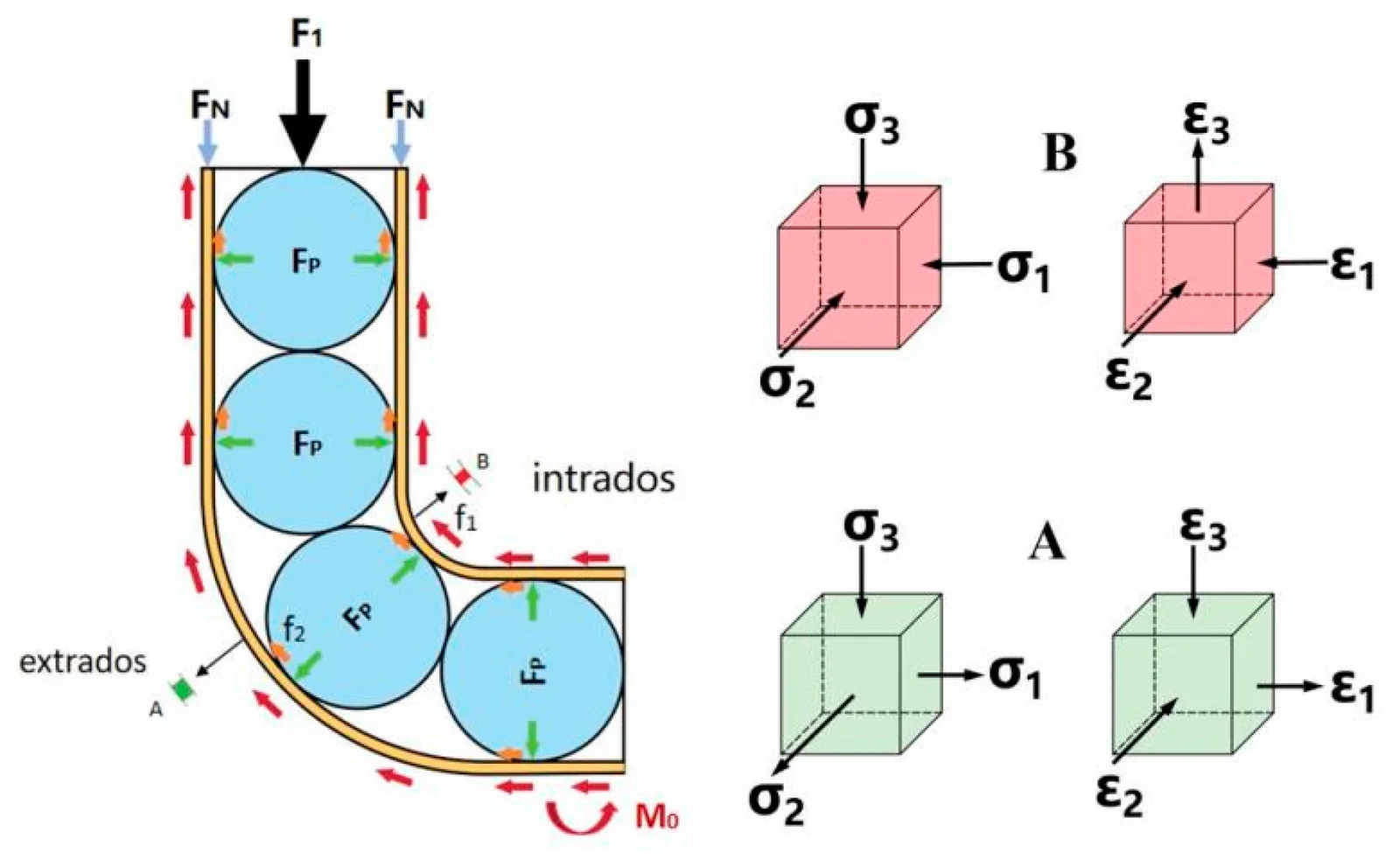
Stress analysis of tube bending.

When the pusher drives the steel balls forward, the steel balls move along a specific radius inside the tube and provide support to the tube. The external forces acting on the tube during push-bending are shown in Figure 2. The stress state of the tube in push-bending differs from that in conventional tube bending. F1 denotes the thrust force, which mitigates wall thinning on the outer bending side. f1 is the friction force between the tube and die, and f2 is the friction force between the tube and steel balls. These friction forces hinder material flow, especially on the inner bending side. FP stands for the supporting force from steel balls, which suppresses tube wrinkling.

As illustrated on the right side of Figure 2, a differential element is adopted to analyze the stress and strain state of the tube blank. A local cylindrical coordinate system is established to guarantee rigorous mechanical treatment. Considering the symmetry of the push-bending process and the thin-wall condition (t/D ≤ 0.05), the shear stresses on the longitudinal and circumferential planes are neglected as infinitesimal quantities of the second order. Accordingly, the model is simplified to a three-principal-stress state. Herein, σ_1_, σ_2_, σ_3_ represent the longitudinal, circumferential and radial principal stresses, respectively, while ε_1_, ε_2_, ε_3_ denote the corresponding principal strains. Specifically, the element at Point B (inner bending side) is mainly subjected to longitudinal compressive stress (σ_1_ < 0), whereas the element at Point A (outer bending side) bears longitudinal tensile stress (σ_1_ > 0). The radial stress σ_3_ induced by the contact pressure from steel balls provides a critical reaction force to resist buckling instability on the inner bending side and improves the stress state of the bent tube.

## 3. Experimental Materials and Methods

### 3.1. Experimental Materials

**Figure 3:**
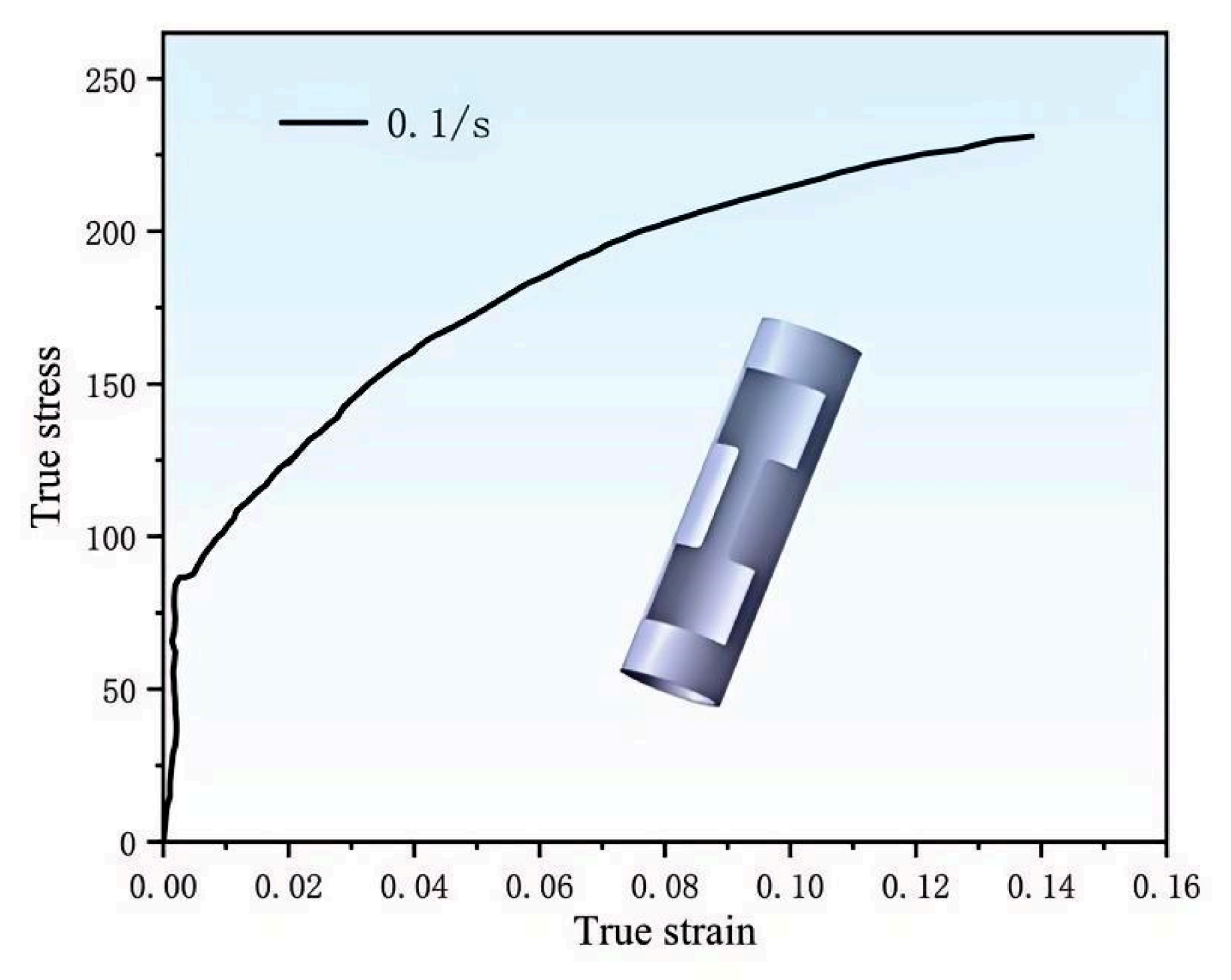
5A02 aluminum alloy stress-strain curve.

The 5A02 aluminum alloy tube used in this experiment has an outer diameter D = 32 mm and wall thickness t = 1 mm. The specimen dimensions are designed according to the tensile test standard GB/T 228.1-2010 [24]. Tensile tests are carried out on a CMT4104 electronic universal testing machine (MTS Industrial Systems (China) Co., Ltd., Shenzhen, China) with a maximum load capacity of 10 kN, manufactured by Shenzhen SANS Material Test Co., Ltd. The elongation of the gauge section of the tensile specimen is measured by a contact extensometer (MTS Industrial Systems (China) Co., Ltd., Shenzhen, China) with a gauge length of 50 mm. To improve the parameter accuracy, three repeated tensile tests are performed under identical conditions, and the average values are adopted. First, the load–displacement curves obtained from experiments are converted into engineering stress–strain curves, and further transformed into true stress–true strain curves, as shown in Figure 3. The basic mechanical properties of the 5A02 aluminum alloy tube at room temperature are calculated and summarized in Table 2. The material properties of G10 bearing steel balls are listed in Table 3.

### 3.2. Geometric Characteristics of Target Parts

**Figure 4:**
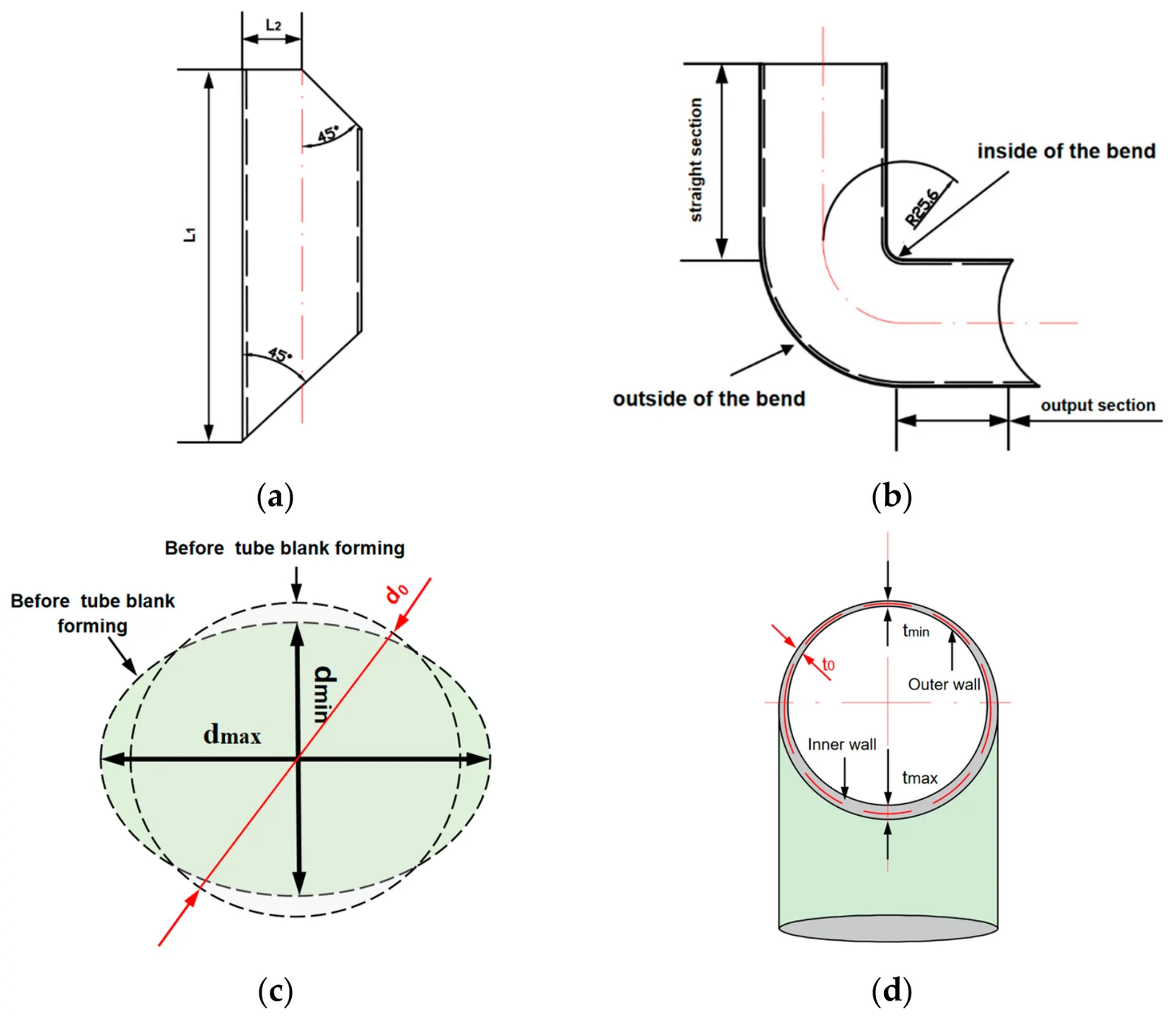
Geometric characteristics of target parts. (**a**) Original size of billet, (**b**) formed fitting, (**c**) diameter of the pipe before and after bending, (**d**) wall thickness parameter of bent tube.

The geometric dimensions of the target tube are shown in Figure 4. Figure 4a presents the structural dimensions of the original tube blank. The ends of the tube blank, with a guiding length L_1_ of 100 mm, are beveled at 45° to offset the volume of material accumulation on the inner side during formation and prevent excessive thickening. The straight section length L_2_ is set to 16 mm. The outer diameter D of the bent tube is 32 mm, the wall thickness t_0_ is 1 mm, and the relative bending radius R is 0.8 D. Figure 4b shows the formed tube with a bending angle of 90° and a bending radius of 25.6 mm.

The ovality and wall thickness distribution are shown in Figure 4c. The ovality and wall thickness distribution of the thin-walled tube before and after deformation are shown in Figure 4d. The maximum ovality γ, maximum thickening rate δ, and maximum thinning rate *α* can be calculated by Formula (1).

(1) $$\begin{array}{c} \gamma =\frac{d_{\max}-d_{\min}}{d_{0}}\times 100\%\\ \delta =\frac{t_{\max}-t_{0}}{t_{0}}\times 100\%\\ \alpha =\frac{t_{0}-t_{\min}}{t_{0}}\times 100\% \end{array}\;$$

In the formula, *t*_0_ and *d*_0_ represent the initial wall thickness and outer diameter of the tube. *t*_max_ and *t*_min_ are the maximum and minimum wall thicknesses of the bent tube, and *d*_max_ and *d*_min_ are the maximum and minimum diameters of the bent tube’s axis.

### 3.3. Forming Equipment and Moulds

**Figure 5:**
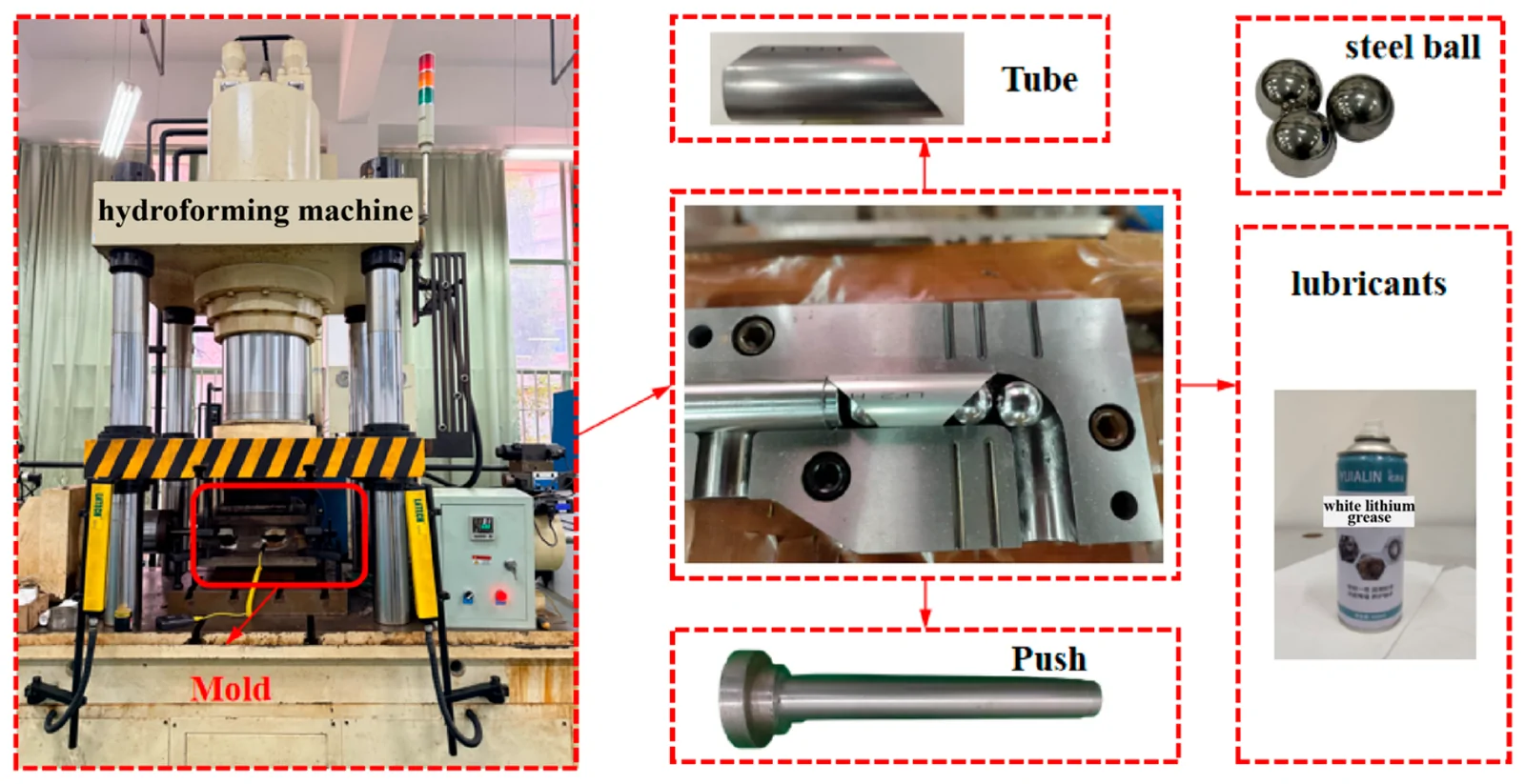
Bending forming equipment and device.

The forming equipment (Foshan Xingdi Hydroforming Equipment Co., Ltd., Foshan, China) adopted in this experiment is shown in Figure 5. Its PLC control system allows the thrust, stroke and feed speed of each cylinder to be set on the operation panel, and the operating status is displayed in real time on the panel screen. This facilitates the regulation of process parameters for small-radius bending of aluminum alloy tubes, as illustrated in Figure 5. The die set for small-radius bending tests of aluminum alloy tubes mainly consists of an upper die, a lower die and a pusher. Before forming, differentiated lubrication schemes are applied for different contact interfaces. For the tube–die cavity interface, three lubrication states, i.e., molybdenum disulfide grease, lithium-based grease and dry friction, are adopted according to test cases, corresponding to different friction levels. By contrast, molybdenum disulfide grease is uniformly applied to the tube–steel ball and steel ball–steel ball interfaces. The lubrication conditions for these interfaces remain constant across all tests and do not vary with orthogonal cases.

## 4. Finite Element Modeling and Verification

A finite element model for steel-ball-assisted push-bending is established on the ABAQUS/Explicit (2021) platform. The 5A02 tube blank is modelled as an isotropic elastoplastic material with a density of 2680 kg/m^3^, an elastic modulus of 70 GPa and a Poisson’s ratio of 0.33. Isotropic hardening is incorporated into the model to characterize the plastic flow during multi-pass bending. Contact interactions are defined by the penalty friction formulation. Three contact pairs are specified separately: tube–die, tube–steel ball and steel ball–steel ball. The friction coefficient of the tube–die contact is taken as factor C in the orthogonal test with three levels of 0.05, 0.10 and 0.15. The tube–steel ball friction coefficient is fixed at 0.12, and the steel ball–steel ball friction coefficient is fixed at 0.10, to mimic the dynamic sliding interface between the tube blank and die.

The three friction levels for the tube–die interface are selected based on experimental experience of similar lubrication tests for aluminum alloy push-bending and published literature on metal plastic forming. The friction coefficients of 0.12 for tube–steel ball contact and 0.10 for steel ball–steel ball contact are empirical engineering values under molybdenum disulfide lubrication, consistent with the constant lubrication state of steel-ball interfaces in physical tests. In the simulations, only the tube–die friction coefficient is varied as the orthogonal variable; the tube–steel ball and steel ball–steel ball friction coefficients remain unchanged across all simulation cases, which matches the lubrication strategy adopted in physical experiments.

For discretization, the tube blank is meshed using four-node reduced-integration curved shell elements (S4R) with hourglass control. The final finite element model of the tube blank contains 6732 nodes and 6565 quadrilateral elements, which provides sufficient resolution to capture local deformation and wrinkling behavior [18]. A prescribed velocity is applied to the pusher along the Y-axis to drive the tube through the die. The die is fully fixed by constraining all degrees of freedom. ZSYMM symmetry boundary conditions (U3 = UR1 = UR2 = 0) are imposed on the tube blank and discretely distributed steel balls. General contact interactions are defined between the inner wall of the tube blank and steel balls, as well as between adjacent steel balls [18]. The modelled tube has an outer diameter of 32 mm, a wall thickness of 1 mm, and a relative bending radius of 0.8 D.

**Figure 6:**
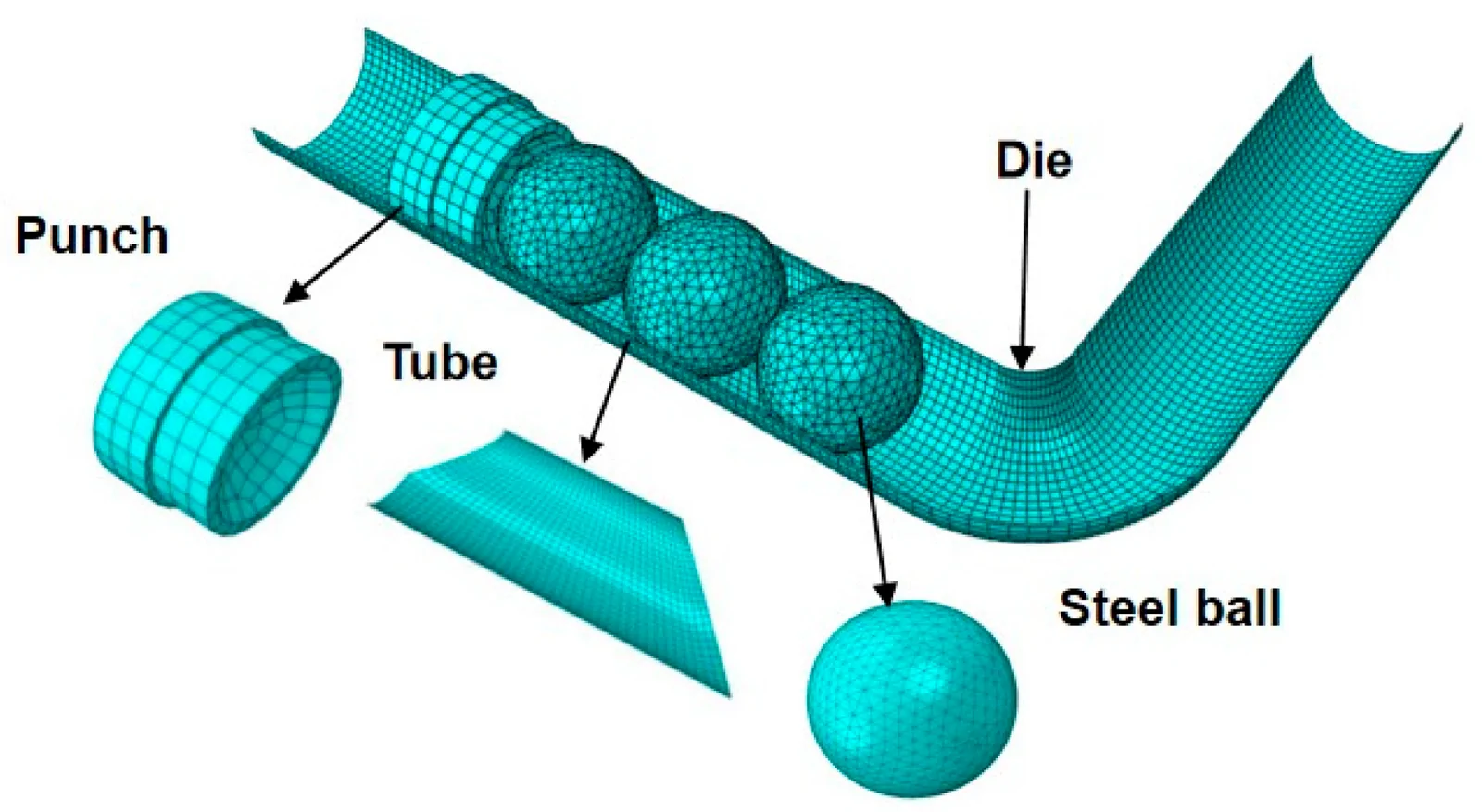
Finite element model.

The geometry, boundary loading conditions, and steel-ball arrangement of the push-bending model are symmetric about the middle parting plane. The thrust acting on the tube, die constraints and internal supporting forces from steel balls satisfy planar symmetry, and no lateral asymmetric external load exists. Given such geometric and loading symmetry, a 1/2 symmetric model is adopted with ZSYMM boundary conditions (U3 = UR1 = UR2 = 0). This approach reduces the model size and shortens computation time while preserving computational accuracy. The symmetry assumption neglects weak asymmetric effects induced by minor random misalignment and assembly deviation of steel balls in real tests, and such asymmetric factors are included in the error sources between simulation and experiment. The simplified model mainly consists of four parts: cavity die, pusher, tube blank, and steel balls, as shown in Figure 6.

The ABAQUS/Explicit solver is used to simulate quasi-static push-bending. A constant feed velocity of 700 mm/s is assigned to the pusher, and the total step time is 0.1 s. Mass scaling is not activated in this model. Energy checks are performed by outputting history variables of kinetic and internal energy. The maximum kinetic-to-internal energy ratio throughout the forming process is less than 5%, so dynamic inertial effects can be neglected, and the quasi-static simulation requirement is satisfied.

Experiments on aluminum alloy tube push-bending were carried out to verify the reliability of the finite element model, with experimental conditions consistent with those adopted in simulations. Three repeated push-bending tests were performed under identical process parameters. All three specimens achieved successful push-bending at 0.8 D without fracture or obvious wrinkling. One specimen was selected for quantitative measurements of wall thickness and cross-sectional ovality, while the other two were used for macroscopic defect observation to demonstrate the reproducibility of the proposed process.

**Figure 7:**
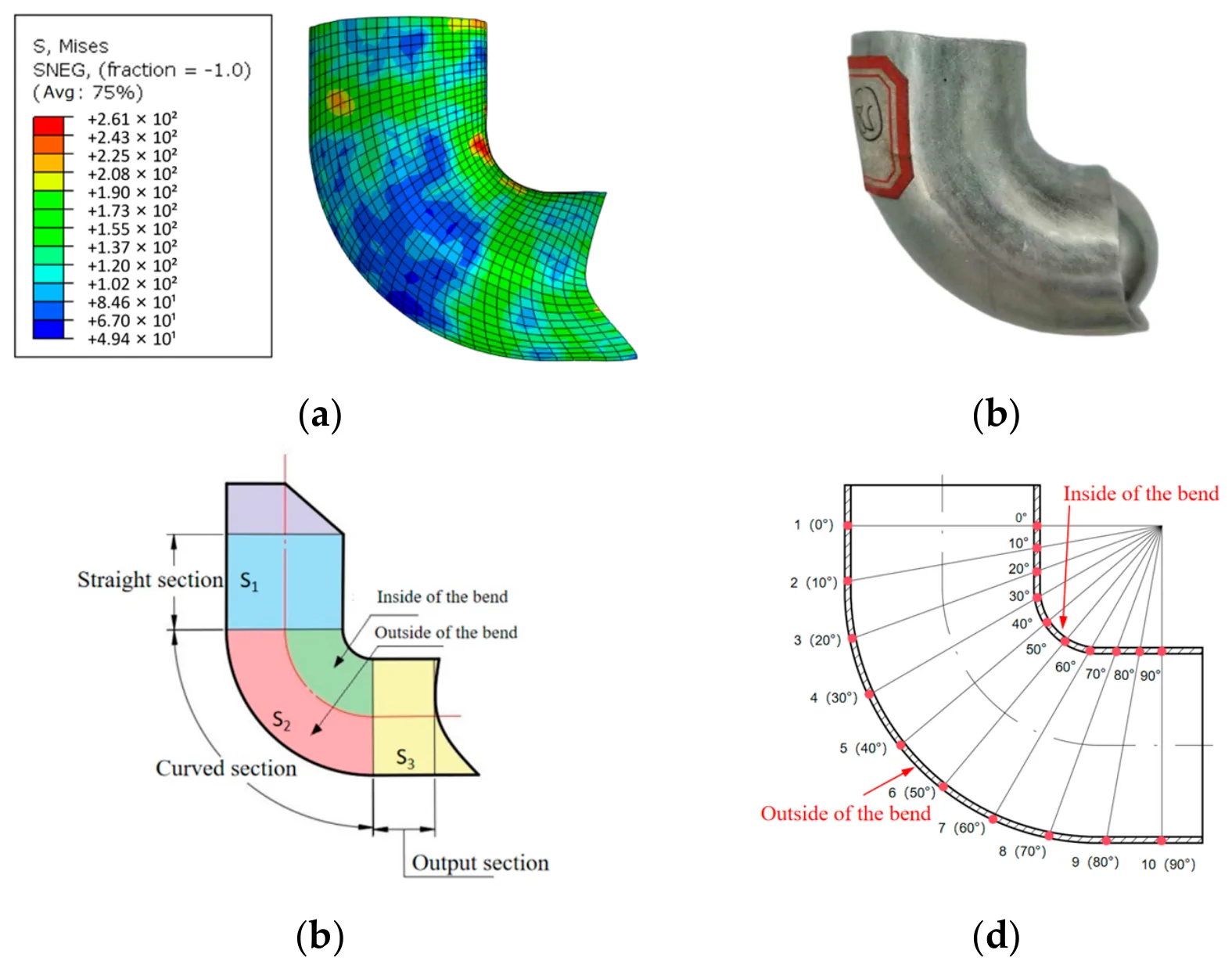
Bent tube formed part and inner and outer wall thickness measurement points. (**a**) Simulated forming tube bending, (**b**) experimental tube bending, (**c**) area division of bent pipe fittings, (**d**) measurement points for wall thickness on the inner and outer sides of a bent tube forming part drawing.

**Figure 8:**
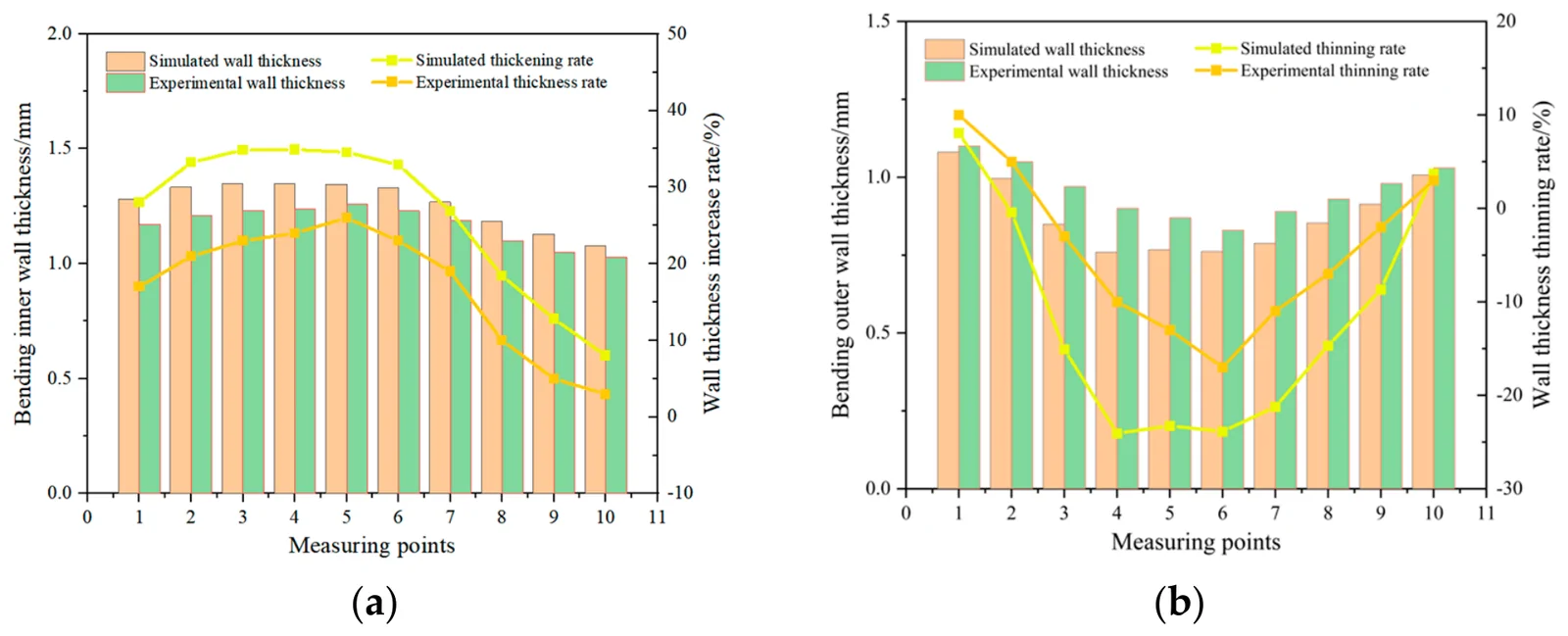
Comparison of inner and outer wall thickness on bending side between simulation and experiment. (**a**) Wall thickness and thickening rate at the inner side of the bend. (**b**) Wall thickness and thinning rate at the outer side of the bend.

For comparison of wall thickness between simulation and experiment, the formed bent tube is shown in Figure 7a. Measurement points were arranged every 10° along the bending zone, as illustrated in Figure 7b. As shown in Figure 8a, the wall thickness on the outer bending side decreases due to longitudinal tension. Nevertheless, the tensile deformation is compensated by axial thrust, and the thinning rate is effectively controlled below 15%. In Figure 8b, the wall thickness on the inner bending side increases remarkably, which originates from the combined effects of bending compression and axial thrust. The smooth curves in both figures indicate that discrete steel balls provide stable internal support, suppressing cross-sectional distortion and ensuring a uniform transition of wall thickness. According to Figure 8, the relative error between experimental and simulated inner-side thickening rates is 11.87%, and the relative error for outer-side thinning rates is 14.07%. The experimentally measured cross-sectional ovality of the bent tube is 2.2%, while the corresponding numerical prediction is 5.2%.

The acceptance criteria for model errors are defined in this work as follows. The model is mainly used to compare the relative variation of forming indicators under different process parameters and identify the primary and secondary influencing factors. For wall-thickness indicators, a relative error within 15% enables satisfactory reproduction of the evolution trend of wall thickness along the bending arc. For cross-sectional ovality, the simulation considerably overestimates the value; high-precision prediction of absolute ovality cannot be achieved, and the absolute ovality values output by this model are not recommended as direct engineering evaluation criteria.

The overall trends of wall thickness along the bending arc obtained from simulation and experiment are consistent. For wall thickness, the relative error of inner-side thickening (11.87%) and outer-side thinning (14.07%) falls within the acceptable range for simulations of this ultra-small-radius push-bending process. However, a notable discrepancy exists in ovality prediction: the maximum simulated ovality is 5.2%, compared with the experimental value of 2.2%. Multiple factors contribute to this deviation. Steel balls in the simulation are ideal geometries with no assembly gaps between them and can deliver continuous and uniform rigid support. In practice, steel balls have manufacturing tolerances, and tiny gaps and local misalignment occur after filling, leading to weaker internal support than in the ideal simulation. In addition, a constant friction coefficient is assigned in the simulation, whereas the lubricant film thickness cannot be perfectly uniform in physical tests. Moreover, the material constitutive relation identified from uniaxial tensile tests cannot fully reproduce the multiaxial stress state during bending, and die assembly gaps also introduce extra errors. Therefore, the finite element model can reliably reproduce the evolution trend of wall thickness, but it markedly overestimates the absolute value of ovality. It is more suitable for comparing relative trends under different process parameters rather than precisely predicting the absolute ovality.

Full circumferential contour scanning of the tube was not performed in the present tests. Instead, discrete measurement points on multiple cross-sections were adopted to obtain ovality at different bending positions and compare the ovality evolution trends along the bending arc between simulation and experiment. Both types of errors are within acceptable limits, which demonstrates the reliability of the finite element model [25].

Finite element simulations were performed for two-pass, three-pass, four-pass, and five-pass push-bending of the tube. The forming quality of bent tubes obtained with different pass numbers was evaluated, and the optimal forming pass number was finally determined according to the best forming performance. Four forming schemes were designed for thin-walled tubes with small relative bending radii. The total feed displacement of the pusher was set to 70 mm for all schemes, and the forming process was divided into from two to five passes. The detailed forming schedules are listed in Table 4.

**Figure 9:**
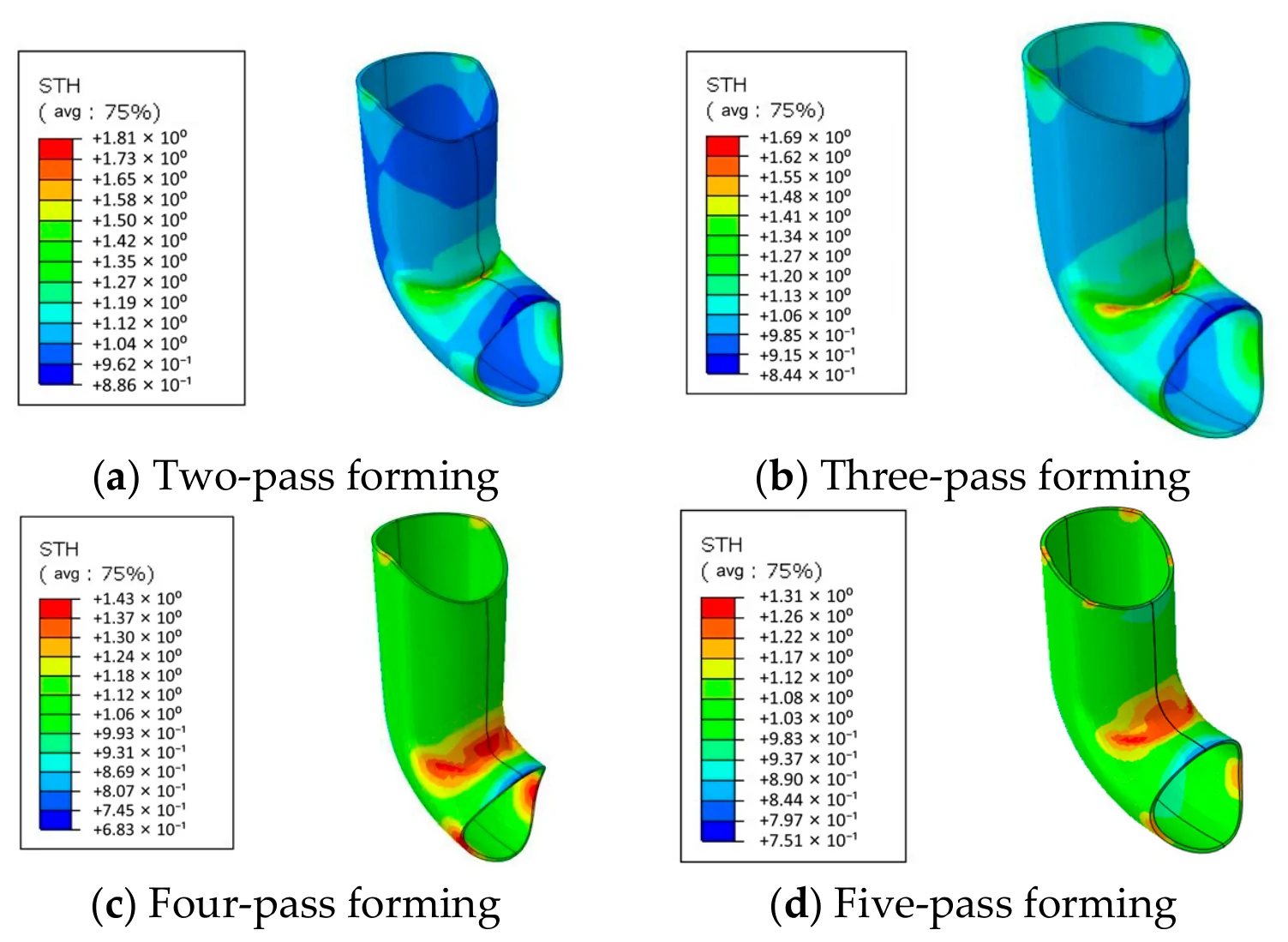
Contours of wall thickness distribution for bent tubes formed with different pass numbers. (**a**) Two-pass forming, (**b**) three-pass forming, (**c**) four-pass forming, (**d**) five-pass forming.

The simulated wall thickness distributions of bent tubes under different forming passes are shown in Figure 9. For two-pass and three-pass forming, severe wall thickening occurs on the inner bending side, which induces wrinkling and uneven wall thickness distribution. Meanwhile, substantial wall thinning at the bending end brings a risk of fracture. The wall thickness distribution of the five-pass bent tube is better than that of the two-pass and three-pass counterparts; nevertheless, severe inner-side thickening remains, accompanied by a wrinkling tendency on the inner bending zone and considerable outer-side thinning with a thinning rate up to 31.8%. The four-pass bent tube exhibits uniform wall thickness distribution, a smooth surface without obvious defects, and the minimum peak stress of 254.2 MPa. It can be observed that increasing the number of forming passes remarkably alleviates inner-side thickening and stress concentration on the inner bending side, gradually improves forming quality and enhances the formability of bent tubes to a certain extent.

For the multi-pass push-bending of thin-walled aluminum alloy tubes with small relative bending radii, the correlation between forming pass number and forming accuracy is positive but nonlinearly convergent. Higher forming accuracy can be achieved with more passes. However, additional passes aggravate work hardening of the material and reduce processing efficiency. Verification shows that the four-pass formed part satisfies practical engineering requirements in terms of dimensional accuracy and surface quality. Therefore, no further passes are adopted, and the steel-ball-assisted four-pass push-bending process is selected.

## 5. Process Parameter Research

### 5.1. Experimental Scheme and Simulation Results

Three major factors affecting tube bending are selected in this study: steel ball diameter (A), tube blank length (B), and friction between tube blank and die cavity (C) [26]. Each factor is assigned three levels. An L9 (33) orthogonal test with three factors and three levels is designed, and the factors together with their levels are listed in Table 5. To meet the requirement on error degrees of freedom for analysis of variance (ANOVA), one additional replicate simulation is performed for each simulation case, yielding a total of 18 finite element simulations. Residuals from the replicated simulations are adopted as the error source for ANOVA. All data from this orthogonal test are obtained from deterministic finite element simulations rather than physical experiments. The replicates here refer to repeated numerical calculations under identical process parameters, instead of repeated physical specimens or physical measurements. Minor output differences among replicate simulations are used to construct the error term for ANOVA. Meanwhile, the L9 (3^3^) orthogonal array cannot separate high-order interactions among factors. Accordingly, the indistinguishable interaction effects are pooled into the error term for F-test calculation.

**Figure 10:**
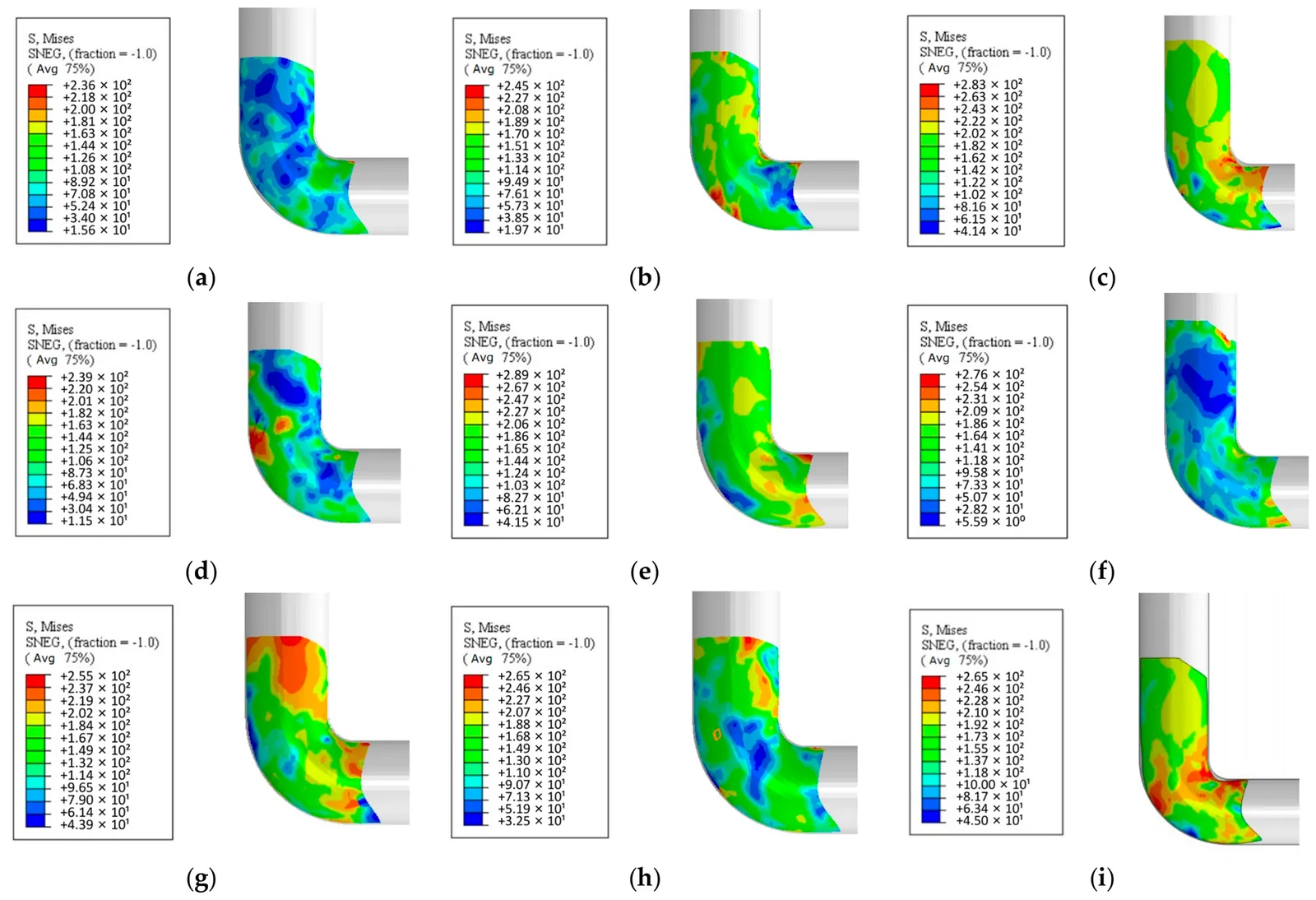
Finite element simulation results. (**a**) Test 1, (**b**) Test 2, (**c**) Test 3, (**d**) Test 4, (**e**) Test 5, (**f**) Test 6, (**g**) Test 7, (**h**) Test 8, (**i**) Test 9.

The experimental design and results are presented in Table 6. Based on the orthogonal experiment, the stress programs for the nine simulated push-bending forming results are depicted in Figure 10. The consistent units of millimeter (mm), ton (t), and second (s) were used, resulting in stress units of MPa.

Obvious stress concentration is observed in specific regions according to the simulation results: the inner and outer bending sides of the bent tube in Figure 10i, the inner bending side in Figure 10c, and the tube end in Figure 10g. Such stress concentrations indicate that, under the corresponding process parameters, the tube blank tends to suffer excessive thickening and even wrinkling instability on the inner bending side. In contrast, the stress gradient distribution in Figure 10e is relatively uniform, suggesting a low wrinkling risk under this set of process conditions. For Figure 10a,b, although the overall stress level is relatively low—which is generally favorable—the use of small-diameter steel balls increases the ovality of the tube and degrades forming accuracy. On the other hand, Figure 10d,f exhibits a more balanced stress distribution on both inner and outer bending sides of the bent tube, accompanied by more uniform wall thickness variation, which reflects satisfactory forming quality.

### 5.2. The Law of Wall Thickness Variation on the Inner and Outer Sides

The wall thickness evolution during 0.8 D small-radius push-bending is not governed by a single factor but dominated by the synergistic interaction among friction coefficient, steel-ball diameter and tube feed length. The analysis reveals that the interaction between friction coefficient and steel-ball diameter plays a critical role in determining the metal flow behavior. A larger steel-ball diameter can provide the required radial pressure to suppress cross-sectional ovalization, yet it also enlarges the contact area. When combined with a high friction coefficient (e.g., f = 0.15), the frictional resistance generated at the tube–steel-ball interface hinders the axial feed force. Consequently, the material on the outer arc side undergoes excessive longitudinal tension and triggers localized thinning.

Furthermore, the tube feed length amplifies this effect. For a longer tube blank, the cumulative frictional resistance along the guiding section increases the required thrust. When a closely fitted steel-ball diameter is adopted, the material on the inner arc side may experience premature thickening or even buckling before entering the bending zone. Therefore, to fabricate an ideal bent tube at 0.8 D, a precise balance must be achieved: optimized lubrication is used to maintain a low friction coefficient, while a steel-ball diameter is selected that can provide sufficient internal support without introducing excessive dragging resistance. Such synergistic control is essential to guarantee uniform plastic strain distribution and preserve the structural integrity of the bent tube.

**Figure 11:**
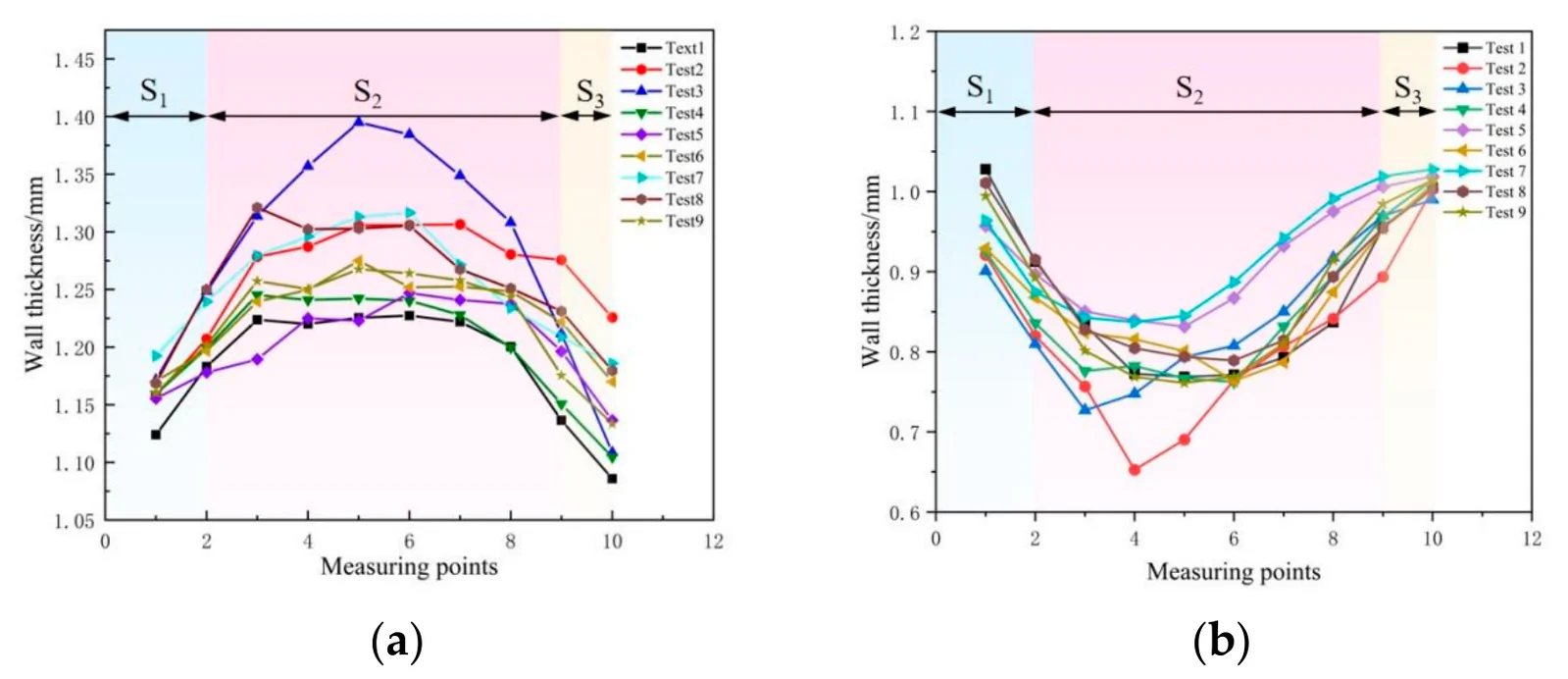
Inner and outer wall thickness: (**a**) simulate the thickness of the inner side wall, (**b**) simulate the thickness of the outer side wall.

Based on finite element simulations, measurement points were arranged every 10° along the bending zone (consistent with the model validation simulation), and the inner and outer wall thickness distributions of bent tubes in the nine simulation groups were measured, as shown in Figure 11a. The overall trend of inner-side wall thickness rises first and then falls, and the maximum thickening rate generally occurs from within 25° to 60° of the bending zone. The inner-wall thickening rate of the third simulation group reaches up to 39.4%. Figure 11b presents the outer-side wall thickness distribution curves, which generally decrease first and then increase. The maximum thinning rate normally appears from 25° to 50° of the bending zone. The second simulation group exhibits the most severe outer-wall thinning, with a maximum thinning rate of 34.74%.

### 5.3. Range Analysis

Range analysis calculates the average indicator values k1, k2, k3 for each factor at different levels. The optimal level is selected according to the engineering requirements of forming indicators. The outer-wall thinning rate, inner-wall thickening rate and cross-sectional ovality are all negative indicators, i.e., lower values are preferred. Thus, the level corresponding to the minimum k value is taken as the preferred level for each indicator. The range R reflects the influence magnitude of the factor on the indicator; a larger R indicates a stronger factor effect.

It should be noted that the maximum outer-wall thinning rate, maximum inner-wall thickening rate, and cross-sectional ovality are conflicting forming objectives. Parameter settings that suppress outer-wall thinning may aggravate inner-side thickening or increase cross-sectional oval distortion. Meanwhile, process conditions that reduce ovality may worsen outer-side thinning. Accordingly, no single set of parameters can simultaneously achieve the individual optimum for all three indicators.

**Figure 12:**
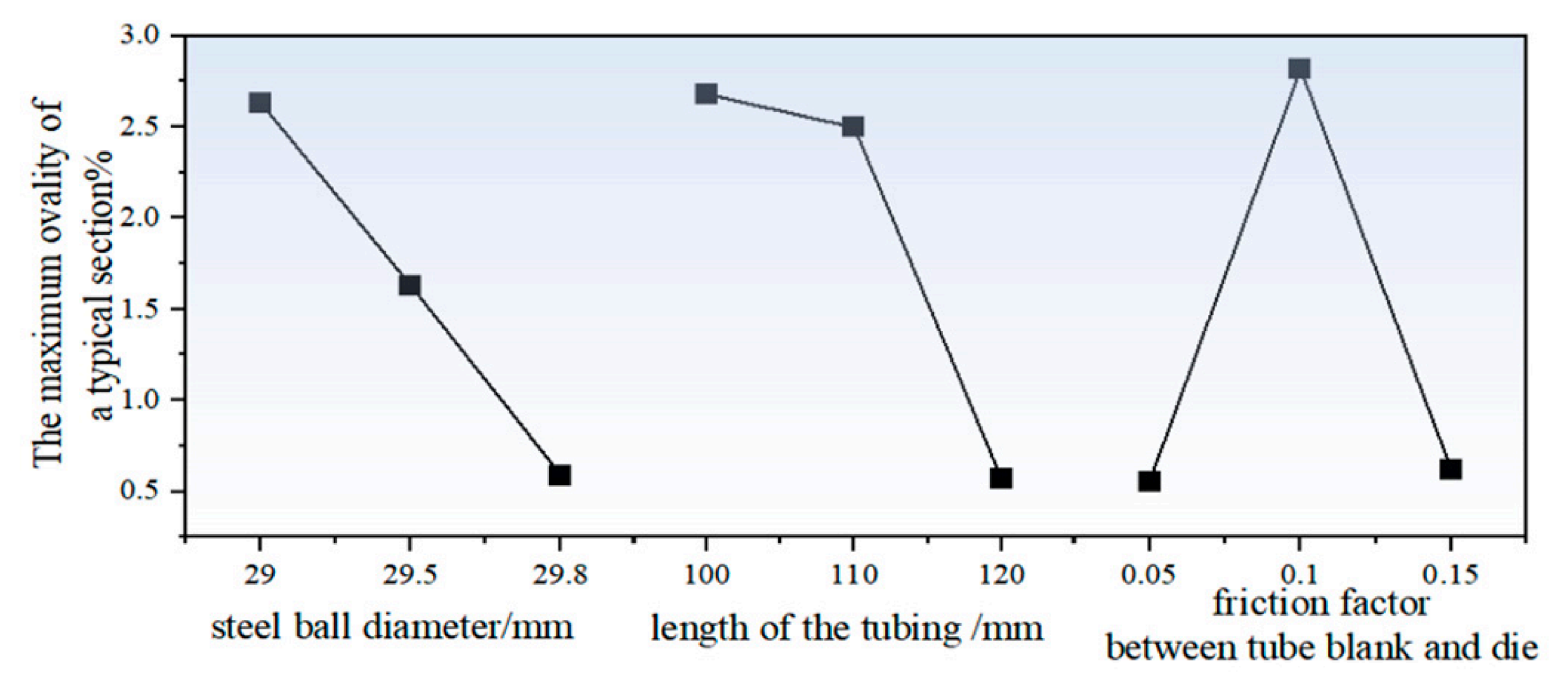
Effect of different factors on the maximum ovality of the typical section.

**Figure 13:**
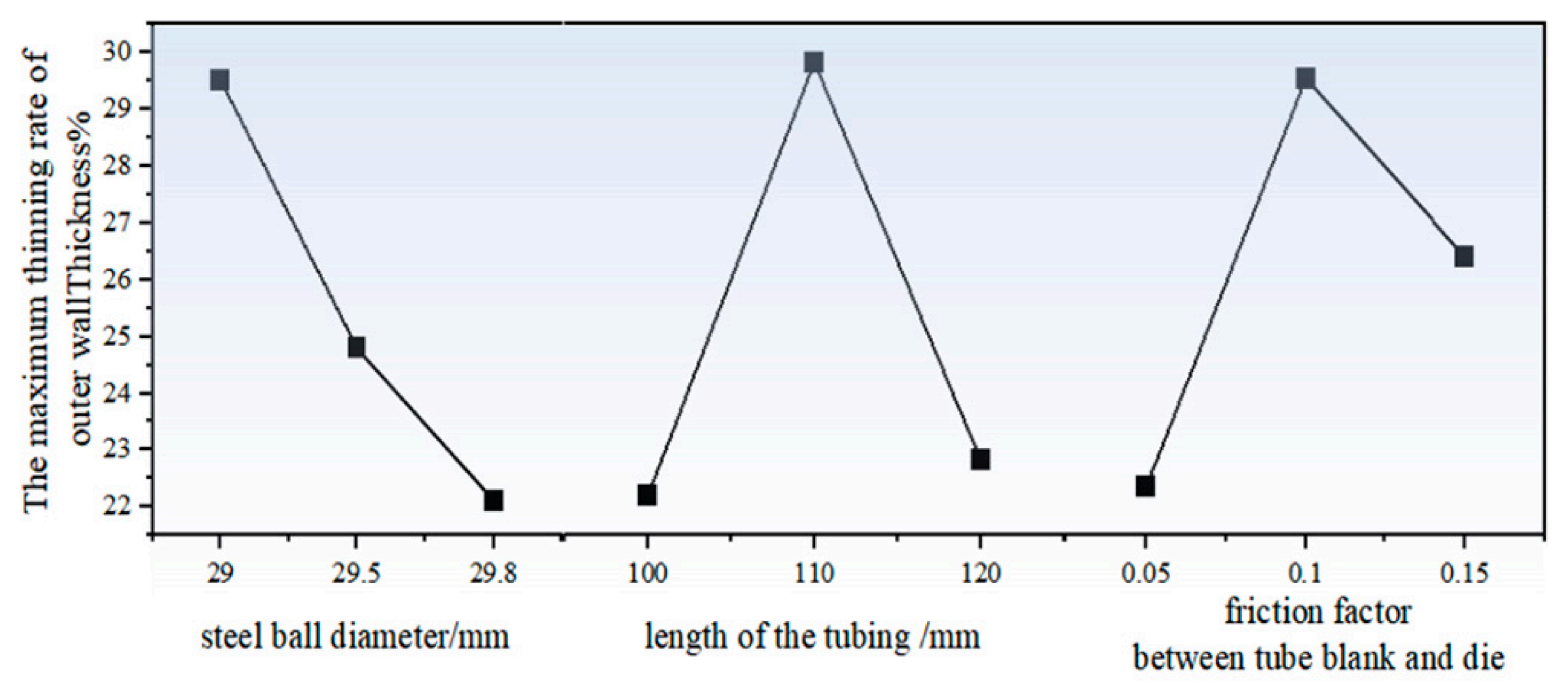
Effect of different factors on the maximum thinning rate of outer wall Thickness%.

From the analysis based on Table 7, Table 8 and Table 9 and Figure 12 and Figure 13, factor A (steel-ball diameter) exerts the most significant effect on the outer-wall thinning rate and the second-greatest effect on the maximum ovality of bent tubes. Its preferred level consistently remains A3, so A3 is selected as the optimal level for factor A. Factor B (tube blank length) has relatively weak influences on ovality and outer-wall thinning rate. B1 performs better in suppressing excessive inner-wall thickening, and hence B1 is adopted as the suitable level for factor B. Factor C (friction between tube blank and die cavity) ranks second and first in its influence on ovality and outer-wall thinning rate, respectively. Its preferred level is consistently C1, showing good balance in mitigating the above two types of forming defects. Therefore, A3 is selected as the preferred level for the single indicator of outer-wall thinning rate. Based on the single-objective evaluation of outer-wall thinning rate, the preferred parameter combination under this single target is A3C1B1.

From the analysis based on Table 7, Table 8 and Table 9 and Figure 12 and Figure 13, factor A (steel-ball diameter) exerts the most significant effect on the outer-wall thinning rate and the second-greatest effect on the maximum ovality of bent tubes. Its preferred level consistently remains A3, so A3 is selected as the optimal level for factor A. Factor B (tube blank length) has relatively weak influences on ovality and outer-wall thinning rate. B1 performs better in suppressing excessive inner-wall thickening, and hence B1 is adopted as the suitable level for factor B. Factor C (friction between tube blank and die cavity) ranks second and first in its influence on ovality and outer-wall thinning rate, respectively. Its preferred level is consistently C1, showing good balance in mitigating the above two types of forming defects. Therefore, A3 is selected as the preferred level for the single indicator of outer-wall thinning rate. Based on the single-objective evaluation of outer-wall thinning rate, the preferred parameter combination under this single target is A3C1B1.

For the maximum thinning rate, factor A (steel-ball diameter) yields the highest F-value (18.54) and the lowest *p*-value (0.002), indicating that it is the most significant influencing factor. This is because the steel-ball diameter directly determines the internal supporting pressure and contact area on the inner bending side, which are critical to the stability of wall thickness during small-radius bending. For cross-sectional ovality, factor C (friction coefficient) exerts the strongest influence, while factor A (steel-ball diameter) and factor B (tube blank length) also impose significant effects on ovality. This statistical result highlights the importance of tribological conditions at the tube–die interface; friction substantially affects material flow and the subsequent cross-sectional distortion. The sum of squares of the error terms in both tables is relatively small. Combined with the duplicate simulation scheme (two replicates for each case), it confirms that the observed effects originate from variations in process parameters rather than simulation random noise, satisfying the requirement for uncertainty quantification. Since localized thinning and cracking are catastrophic failure modes that directly compromise structural integrity, a higher weight of 0.7 is assigned to the thinning rate, and a weight of 0.3 to cross-sectional ovality. Under the multi-objective weighted evaluation framework with the 0.7/0.3 weight ratio and the constraint of inner-wall thickening, the comprehensive preferred parameter combination A3C1B3 is obtained. This combination optimizes the geometric accuracy of the bent tube while keeping the maximum thinning rate within the safe limit. It can be seen that A3C1B1 only pursues the performance of outer-wall thinning suppression at the expense of cross-sectional oval accuracy; by contrast, A3C1B3 balances the risk of thinning-induced cracking and cross-sectional geometric accuracy, delivering a multi-objective compromise solution within the feasible region constrained by thickening.

Range analysis performs independent optimization for each single evaluation index. A3C1B1 is the optimal parameter combination obtained solely targeting the maximum outer-wall thinning rate. This combination prioritizes the suppression of outer-side wall thinning without simultaneous constraints on cross-sectional ovality. In comparison, the weighted comprehensive evaluation is a multi-objective trade-off optimization, in which both the thinning rate and ovality are normalized and weighted. It reduces the risk of outer-side cracking while taking cross-sectional geometric accuracy into account. Consequently, the optimal parameter set from the comprehensive evaluation shifts to A3C1B3. The two methods differ in optimization objectives, leading to different optimal parameter combinations.

The maximum inner-wall thickening rate is an important indicator for evaluating the wrinkling risk on the inner tube side and is not discarded in this work. Before weighted comprehensive evaluation, the maximum inner-wall thickening rate is adopted as a feasibility constraint for scheme screening across all nine orthogonal cases. Cases with severe thickening and high wrinkling risk are eliminated. Among all orthogonal cases in this group, the screened feasible schemes exhibit inner-wall thickening below the wrinkling failure threshold. Outer-wall thinning directly causes cracking and scrapping of tubes and represents a rigid failure index. Therefore, the maximum outer-wall thinning rate and cross-sectional ovality are taken as the objective functions for weighted evaluation, whereas the maximum inner-wall thickening rate only serves as a constraint and is not included in the weighted score calculation.

In this study, the maximum outer-wall thinning rate Tr and cross-sectional ovality E are selected as multi-objective evaluation indicators. Both are negative indicators: smaller values correspond to better forming quality. The min–max normalization method is adopted to non-dimensionalize the raw indicators, and the normalization formula is expressed as:

(2) $$x_{i}^{*}=\frac{x_{\max}-x_{i}}{x_{\max}-x_{\min}}$$

where the raw value of the indicator for the i-th orthogonal scheme is denoted; the maximum and minimum values of this indicator across all nine orthogonal cases are given. The normalized value ranges from [0, 1]. A smaller raw indicator value yields a higher normalized score.

The objective function for comprehensive evaluation is written as:

(3) $$S=0.7\cdot T_{r}^{*}+0.3\cdot E^{*}$$

where S denotes the comprehensive forming score; the normalized scores of the maximum outer-wall thinning rate and cross-sectional ovality are given separately; 0.7 and 0.3 are the weight coefficients for the two indicators, respectively. A higher comprehensive score S indicates better overall forming performance, and all schemes are ranked in descending order of S.

It should be noted that both A3C1B1 and A3C1B3 are parameter combinations predicted by the evaluation model based on orthogonal test data. Among them, A3C1B3 is not included in the nine·g to technical specifications of parts to obtain process schemes for different priorities.

Given that localized thinning and cracking are catastrophic failure modes that directly impair structural integrity, the thinning rate is assigned a higher weight of 0.7 and cross-sectional ovality a weight of 0.3. Based on the weighted performance evaluation, the optimal combination A3C1B3 is determined. This combination optimizes the geometric accuracy of the bent tube while confining the maximum thinning rate within the safe limit.

## 6. Experimental Verification

In this study, the friction coefficient f was controlled by varying the lubrication conditions at the interfaces between the tube, dies, and steel balls. Specifically, Level 1 (f = 0.05) was achieved by applying a thick, uniform layer of high-performance molybdenum disulfide (MoS_2_) grease, which was reapplied before each pass to ensure stable boundary lubrication. Level 2 (f = 0.10) was realized using white lithium grease applied with a standardized spreading tool to maintain a consistent mixed lubrication state. Level 3 (f = 0.15) represented the maximum friction condition (starved lubrication), which was achieved under dry friction by maintaining all contact surfaces in a clean and dry state without the application of any lubricant.

Physical push-bending verification tests were carried out using the preferred parameter combination A3C1B3 obtained from multi-objective weighted evaluation. This parameter combination is an off-table optimal scheme predicted by the orthogonal model and is not among the nine simulation cases of the L9 orthogonal design in Table 5. The nine orthogonal simulation cases in Table 5 cover a wide range of process parameters, including several harsh forming conditions. Accordingly, the distribution range of the maximum outer-wall thinning rate in Table 6 is 20.6–34.74%, which cannot be directly compared with the present verification test.

Certain discrepancies still exist between simulation and experiment, and the error sources are consistent with those discussed in the model validation section of Chapter 4. Ideal steel-ball geometry and constant friction coefficients are adopted in the simulation, while practical factors such as assembly gaps between steel balls, non-uniform lubricant coating, material anisotropy of the tube, and die assembly gaps are neglected. As a result, the predicted thinning and thickening values from simulation are slightly higher than the experimental measurements. The evolution trends of wall thickness along the bending arc obtained from the experiment and the supplementary simulation of A3C1B3 are consistent, further confirming that the model is suitable for evaluating the relative effects of process parameters.

**Figure 14:**
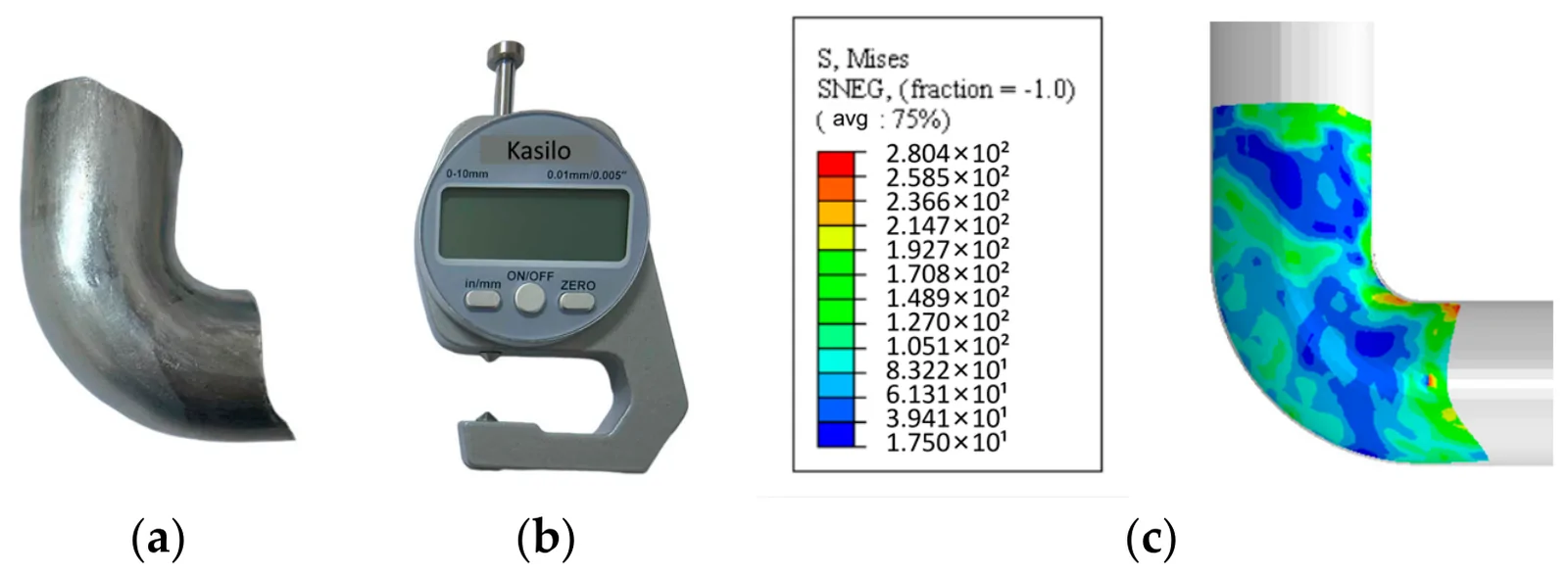
Forming results, thickness gauge and simulation of forming results. (**a**) Forming results, (**b**) wall thickness gauge, (**c**) simulation of forming results.

**Figure 15:**
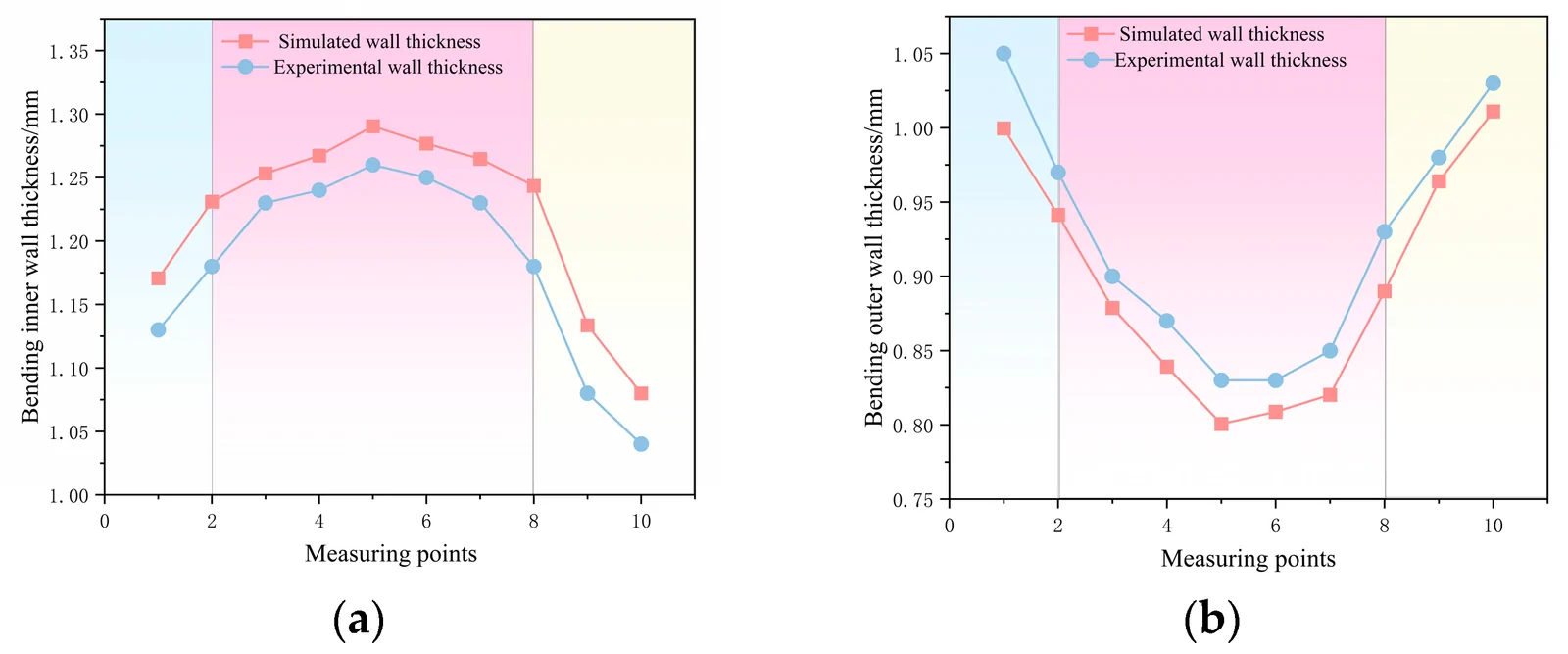
Comparison of simulation and experimental results. (**a**) Inner curve, (**b**) outer curve.

As shown in Figure 14, cross-sectional distortion of the bent tube is largely eliminated, no obvious stress concentration appears in the bending zone, and the wall thickness distribution on both inner and outer sides becomes more uniform. The formed bent tube was equally divided into ten segments to set measurement points (consistent with the validation simulation), and wall thickness was measured by a thickness gauge (Kasilo, Shangqiu, China), as shown in Figure 14b. The wall thickness results are compared with simulation data in Figure 15. A separate supplementary finite element simulation was established for the A3C1B3 parameters adopted in the test. The supplementary simulation yields a maximum outer-wall thinning rate of 15.3% and a maximum inner-wall thickening rate of 28%. In physical tests, the minimum outer wall thickness of the tube is measured as 0.83 mm, corresponding to an outer-wall thinning rate of approximately 17.0%; the maximum inner wall thickness is 1.26 mm, corresponding to an inner-wall thickening rate of approximately 26.0%. The experimental results are basically consistent with the simulation predictions within experimental error.

## 7. Conclusions

(1) The use of discrete steel balls as an internal support medium significantly reduces cross-sectional distortion and maintains high geometric precision. The multi-point dynamic contact between the steel balls and the inner wall provides effective internal pressure, which is crucial for preventing collapse and wrinkling.

(2) Optimization of process parameters, particularly the friction and lubrication conditions, plays a dual role: it not only facilitates a more uniform metal flow to alleviate thinning and thickening but also shows potential to preserve the surface integrity of the aluminum alloy. A moderate friction coefficient at the die interface is expected to mitigate surface galling and micro-crack initiation, which may help maintain substrate quality suitable for subsequent surface engineering processes. It should be noted that these surface-related benefits are inferred from the forming principle and have not been directly verified by dedicated surface tests in this work.

(3) Compared with traditional liquid-filling methods, the discrete steel ball support may reduce surface erosion and internal contamination while providing stable mechanical support. This mechanism is expected to achieve a superior balance between extreme forming limits and surface performance. The potential for favorable surface topography under 0.8 D deformation makes this process highly relevant for aerospace and automotive components requiring subsequent functional coatings or anti-corrosion treatments, providing a reference for interfacial management in high-end manufacturing. Further dedicated surface integrity characterizations, including roughness measurement and microscopic observation, are required to validate the above-mentioned surface-related advantages.

## Figures and Tables

**Table 1:** Comparison of research progress and methods in push bending.

Research Phase/Method	Typical Strategy	Bending Limit R/D	Advantages	Disadvantages	Current
Conventional Push Bending	Rigid die, no internal support	≥2.0 D	Simple structure, high efficiency	Severe thinning, high risk of collapse	Limited to large bend radius
Mandrel-supported Bending	Solid or articulated mandrels	1.0 D–1.5 D	Good ellipticity control for medium bend radius	Interference at small bend radius, tool wear	Failed at 0.8 D due to interference
Granular Media Filling	Sand, beads, or polymers	≥1.0 D	Flexible support, reduced wrinkling	Unstable pressure, hard to clean	Insufficient stability for ultra-small bend radius
Hydroforming	High-pressure liquid	1.2 D–1.5 D	Uniform pressure, excellent surface	Complex sealing, low efficiency	High cost, sealing fails at 0.8 D
Present Study	Discrete Balls + 4-Pass	0.8 D	Rigid support, self-circulation, stress optimization	-	achieve stable 0.8 D bending

**Table 2:** Basic mechanical properties parameters of 5A02 aluminum alloy tube.

Parameter	Yield Strength/ MPa	Tensile Strength/ MPa	Young’s Modulus/ GPa	Density g/cm^3^	Poisson’s Ratio
Value	84	232	70	2.68	0.33

**Table 3:** Material properties parameters of G10 bearing steel ball.

Parameter	Yield Strength/ MPa	Tensile Strength/ MPa	Young’s Modulus/ GPa	Density g/cm^3^	Poisson’s Ratio
Value	1600	2000	210	7.81	0.28

**Table 4:** Multi-pass forming schemes.

	Number of Forming Passes	Pass 1	Pass 2	Pass 3	Pass 4	Pass 5
Forming Scheme						
Two-pass forming		X150	X220			
Three-pass forming		X140	X220	X310		
Four-pass forming		X130	X220	X315	X45	
Five-pass forming		X130	X215	X310	X410	X55

**Table 5:** Factors and levels.

Levels	A, Steel Ball Diameter/mm	B, Length of the Tubing/mm	C, Friction Factor Between Tube Blank and Die
1	29	100	0.05
2	29.5	110	0.10
3	29.8	120	0.15

**Table 6:** Virtual orthogonal test results.

Test Number	A	B	C	The Maximum Thinning Rate of the Outer Wall Thickness%	The Maximum Thickening Rate of Medial Wall Thickness%	The Maximum Ovality of a Typical Section%
1	1	1	1	26.6	22.72	2.94
2	1	2	3	34.74	30.64	2.26
3	1	3	2	27.31	39.4	2.69
4	2	1	3	22.6	24.02	1.8
5	2	2	2	31.2	23.68	2.01
6	2	3	1	20.6	23.63	1.09
7	3	1	2	21.3	31.6	0.61
8	3	2	1	21.09	30.5	0.96
9	3	3	3	23.94	26.8	0.19

**Table 7:** Range analysis results 1.

Index	Parameters	A, Steel Ball Diameter/mm	B, Length of the Tubing/mm	C, Friction Factor Between Tube Blank and Die
The maximum thinning rate of outer wall Thickness%	K1	88.65	70.5	71.14
	K2	74.4	87.03	89.11
	K3	66.33	71.85	81.28
	k1	29.5	23.5	23.71
	k2	24.8	29.01	29.70
	k3	22.11	23.95	27.09
	Range R_g_	7.44	5.51	5.99
	Factors of influence primary and secondary			A > C > B
	Better solution		A3C1B1	

**Table 8:** Range analysis results 2.

Index	Parameters	A, Steel Ball Diameter/mm	B, Length of the Tubing/mm	C, Friction Factor Between Tube Blank and Die
The maximum ovality of a typical section%	K1	7.89	5.35	4.22
	K2	4.9	5.23	5.31
	K3	1.76	3.97	4.25
	k1	2.63	1.78	1.40
	k2	1.63	1.74	1.77
	k3	0.59	1.32	1.41
	Range Rg	2.04	1.37	2.50
	Factors of influence primary and secondary			C > A > B
	Better solution		C1A3B3	

**Table 9:** Analysis of variance results.

Index	Source	SS	df	MS	F-Value	Significance
The maximum thinning rate of outer wall Thickness%	A	32.45	2	16.225	18.54	Significant
	B	14.12	2	7.06	8.07	Significant
	C	16.58	2	8.29	9.47	Significant
	Error	3.5	11	0.875		
	Total	66.65	17			
The maximum ovality of a typical section%	A	8.42	2	4.21	10.65	Significant
	B	4.15	2	2.075	5.25	Significant
	C	12.36	2	6.18	15.65	Significant
	Error	1.58	11	0.395		
	Total	26.51	17			

Note: Significance level α = 0.05; critical value F0.05 (2,11) = 3.98. Each orthogonal scheme contains two repeated simulation runs. The error term consists of residuals from repeated simulations and unidentifiable interaction effects.

## Data Availability

The original contributions presented in the study are included in the article. Further inquiries can be directed to the corresponding author.
